# New Approach to Assembling Nucleic Acid Dendrons on a Solid Phase

**DOI:** 10.1021/acs.orglett.5c02877

**Published:** 2025-08-20

**Authors:** Gerald J. Chik, Afaf H. El-Sagheer, Tom Brown

**Affiliations:** † Department of Chemistry, Chemistry Research Laboratory, 6396 University of Oxford, Oxford OX1 3TA, United Kingdom; ‡ School of Chemistry and Chemical Engineering, 243732 University of Southampton, Highfield, Southampton SO17 1BJ, United Kingdom

## Abstract

We report the methodology to synthesize nucleic acid dendrons on a solid support via Cu­(I)-catalyzed alkyne–azide cycloaddition (CuAAC). Current methods require excess coupling reagents and result in low yields. Our alternative strategy is based on standard solid-phase synthesis followed by 5′-end coupling of a multifunction linker to efficiently afford dendrons. This approach enables the synthesis of DNA/RNA dendrons whose properties are compatible with therapeutic applications.

## Graphical abstract

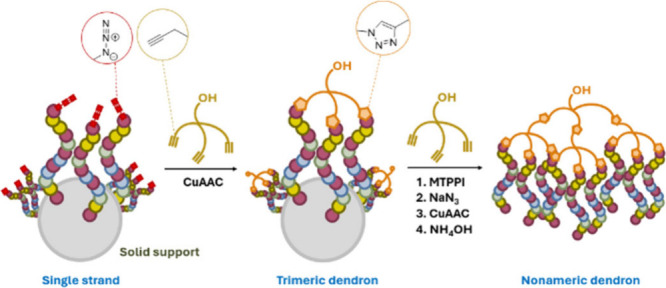

In recent years, nucleic acid dendrons (NADs) have emerged as a new class of functional polymer clusters with a well-defined, brush-like architecture. They are similar to spherical nucleic acids (SNAs) that demonstrate unique properties, such as colloidal aggregation and enhanced cellular uptake.^1–4^ Like SNAs, NADs are composed of oligonucleotide (ON) clusters with a valency defined by the number of ON strands but are conjugated through a single branching unit instead of a three-dimensional core, making them structurally analogous to molecular dendrons.^5,6^

These clusters exhibit significantly different properties than their single-stranded counterparts and have various applications. For example, they act as probes in the polymerase chain reactions (PCRs) as their macromolecular structures increase friction in polymeric gels and lower the electrophoretic mobility of the target amplicon.^7^ Their dense negative charges also reduce accessibility to and inhibit the activity of nuclease enzymes, making them useful in siRNA and nanoparticle scaffolds.^8–10^ In addition to biophysical properties, polyanionic NADs can also trigger scavenger receptor-mediated endocytosis and elicit potent immune responses in vaccine formulations.^11,12^ Recently, NADs have been shown to promote cellular internalization of large proteins when the two molecules are covalently conjugated.^13,14^ Together, these beneficial effects show the promise of NADs as a next-generation therapeutic modality.

Despite their biological relevance and promising applications, the efficient synthesis of NADs remains challenging. While the controlled assembly of oligonucleotides by noncovalent binding to form dendritic structures has been well-documented,^15–19^ methods to synthesize covalently connected NADs are limited. Early attempt to access NADs by solid-phase oligonucleotide synthesis (SPOS) relied on a linker molecule 2′,3′-*O*-bis­(2-cyanoethyl-*N*,*N*-diisopropylphosphoramidite) (**1**) (Figure 1) that reacts with the 5′-hydroxy groups on two oligo strands on a solid support to afford a dimeric NAD. This can be further elongated and coupled to other dendrons in an iterative manner.^20^ The efficiency of this approach, however, is inherently limited by the requirement to bring two 5′-OH groups into proximity of each other to react with **1**. This generates a mixture of byproducts with truncated strands. The development of “trebler” phosphoramidite (**2**) and a solid support (**3**) with three dimethoxytrityl (DMT)-protected hydroxy groups, which can be individually elongated, has enabled the synthesis of NADs from a 3′-branching point. This approach is not limited by a precise monomer concentration or interstrand distance and allows for efficient DMT-on purification.^8^ When the pore size of the solid support, length of the linker, and loading of phosphoramidites were changed, significant yield improvements have been demonstrated on a poly­(thymidine) (T) dendron.^11^ Nonetheless, this strategy requires excess reagent loading and is prone to forming truncated products.^7^ This issue is more pronounced when modified nucleosides are introduced.

**Figure 1:**
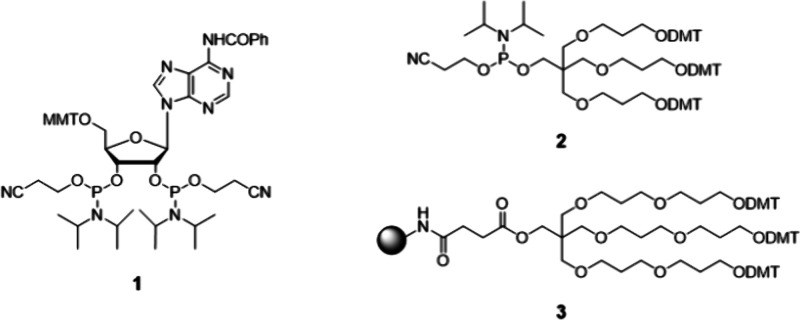
Examples of building blocks in the synthesis of nucleic acid dendrons.

Inspired by previous reports on copper-catalyzed azide–alkyne cycloaddition (CuAAC) to couple nucleosides and oligonucleotides via triazole linkages,^21–27^ we explored the possibility of coupling multiple oligonucleotide strands by a polyvalent alkynyl linker to assemble NADs in a “bottom-up” order on a solid support. Importantly, this strategy requires only standard phosphoramidite coupling conditions for single-strand assembly followed by 5′-azido substitution and click chemistry. This eliminates the use of excess reagents and minimizes coupling failures on multiple strands within a single molecule.

We commenced with the synthesis of a trivalent alkynyl linker, which reacts with three oligonucleotides with 5′-azido modifications (Scheme 1). This linker design incorporates multiple ethylene glycol units to increase flexibility and to accommodate the reactive azide groups at a typical interstrand distance of <25 Å on controlled-pore glass (CPG).^20^ Starting from gallic acid **4a** as a core branching unit, we incorporated alkynyl spacer arm **5a** by treating methyl ester **4b** with 3-(2-(2-iodoethoxy)­ethoxy)­prop-1-yne under basic conditions. Subsequent hydrolysis of the ester yielded compound **5b**, which was coupled with 2-(2-(2-aminoethoxy)­ethoxy)­ethanol to form the linker construct **6** (L3OH) that is stable under standard oligonucleotide deprotection conditions and is soluble in aqueous or organic solvents that support CuAAC.

**Scheme 1:**
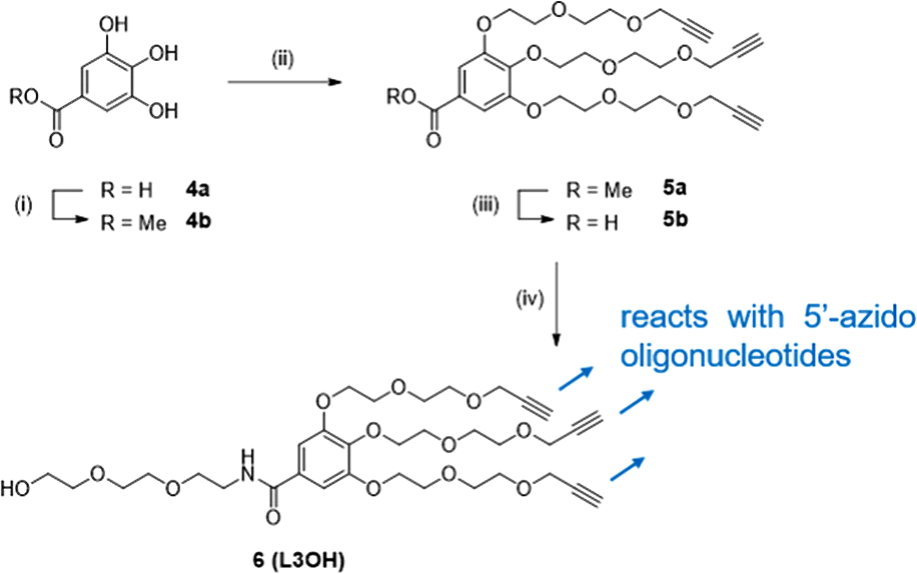
Synthesis of a Trivalent Alkynyl Linker, Which Reacts with 5′-Azido Oligonucleotides Bound on a Solid Support^a^

a (i) Concentrated H_2_SO_4_, methanol, reflux, 18 h; (ii) (3-(2-(2-iodoethoxy)­ethoxy)­prop-1-yne), K_2_CO_3_, DMF, 60 °C, 18 h; (iii) aqueous NaOH, dioxane, 50 °C, 4 h; and (iv) 2-[2-(2-aminoethoxy)­ethoxy]­ethanol, HBTU, DIPEA, DMF, r.t., 24 h.

Next, we explored the feasibility of linking oligonucleotides together via CuAAC on a solid support to yield a trimeric dendron. To this end, a model T_12_ oligonucleotide **ON1** with 5′-OH was synthesized under standard conditions on CPG resin at a 0.2 μmol scale and converted to 5′-azide **ON2** by sequential treatments of methyltriphenoxyphosphonium iodide (MTPPI) and NaN_3_ in DMF (Figure S2).^28,29^ The resin-bound oligonucleotide was then treated with a mixture of L3OH, CuSO_4_–THPTA, and sodium ascorbate and subjected to ammonia treatment to yield the product in solution. Using UPLC–MS characterization, the desired species whose mass corresponds to triply conjugated dendron **ON3** with triazole linkages was identified. A 1 mol equiv of 5′-azido oligonucleotides requires 0.33 mol equiv of L3OH for complete assembly. We used various equivalents of L3OH in the click reaction (0.15, 0.3, and 0.45 mol equiv) to assess product formation when L3OH is limiting or in excess. The percentage conversion of the trimeric dendron improved from 9 to 58% with increasing amounts of L3OH (Figure S3). Partly constructed bis- and monoconjugates were present at less than 5%. The trimeric product is favored due to the close proximity of the alkynyl groups bound to the solid support after the click reaction of L3OH with the first azide.

We then identified the optimal reaction conditions. The observation of fast kinetics of the subsequent CuAAC reactions relative to the first prompted us to use L3OH at controlled concentrations to improve conversion (Figure 2). As expected, at a lower concentration, where 0.25 mol equiv of L3OH was present, a significant ratio of **ON2** remained unconjugated, giving conversions to trimeric dendron from 30 to 54% with 4 and 8 h of reaction, respectively. When the amount of L3OH was increased to 0.5 mol equiv, we observed an 80% conversion for reactions at room temperature (8 h) and 45 °C (4 h), while incomplete conjugates remained at <12%. Encouraged by these results, we synthesized a mixed-base sequence **ON4** on a standard 1 μmol CPG C column and converted it to 5′-azide **ON5** (N3-TCACT­TCGCT­TCAAC).

**Figure 2:**
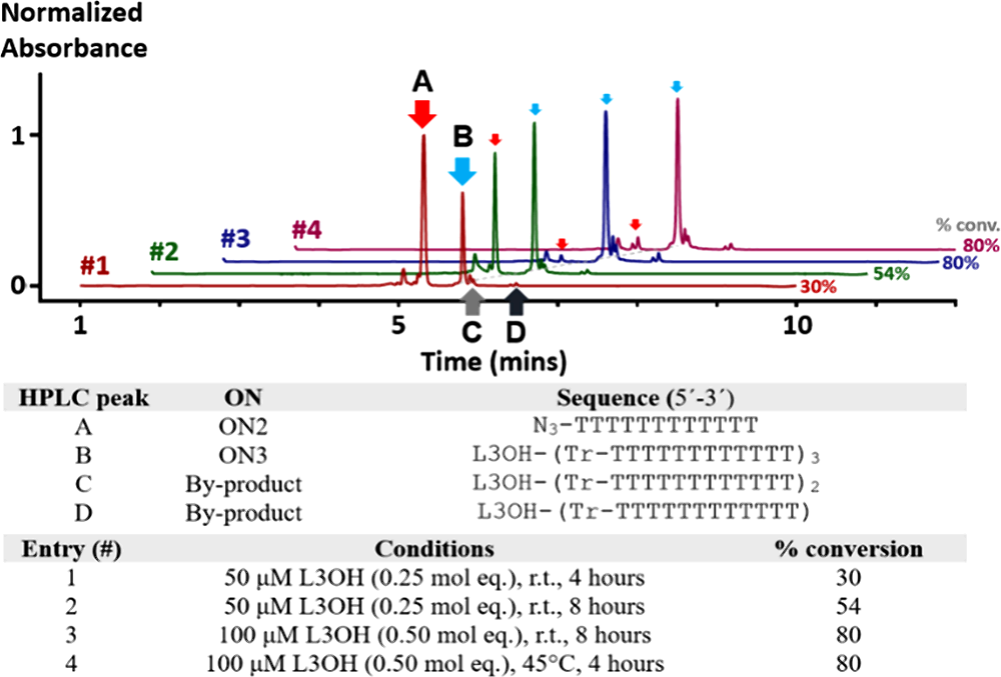
UPLC–MS analysis of T_12_ dendron assembly under various conditions (entries 1–4). CuAAC conditions: 0.5 mM CuSO_4_, 1.0 mM THPTA, and 1.0 mM sodium ascorbate in 100 mM TEAA buffer (20% DMSO, total volume = 1000 μL).

Under optimized click conditions, trimeric dendron product **ON6** was obtained at 80% conversion, whereas the bis-conjugated side product was present at 19% (Figure 3). Next, we explored the assembly of ribonucleosides to form RNA dendrons using 2′-*O*-*tert*-butyldimethylsilyl (TBDMS)-protected ribose. After the click reaction protocol was applied to **ON7** (UCACU­UCGCU­UCAAC), a RNA equivalent of **ON4**, we analyzed the product formed by the deprotection treatment with ammonia and subsequently Et_3_N·HF (Figure S4). The oligoribonucleotide was obtained with a crude purity of ∼98%. Conversion to 5′-azide on the solid support using MTPPI and NaN_3_ gave **ON8** with a conversion of 80%. We then proceeded with the click reaction with L3OH. The conversion to trimeric product **ON9** was 72%, whereas the dimeric byproduct was far less prevalent (11%). These results show that our methodology can be used on oligoribonucleotides to form RNA NADs.

**Figure 3:**
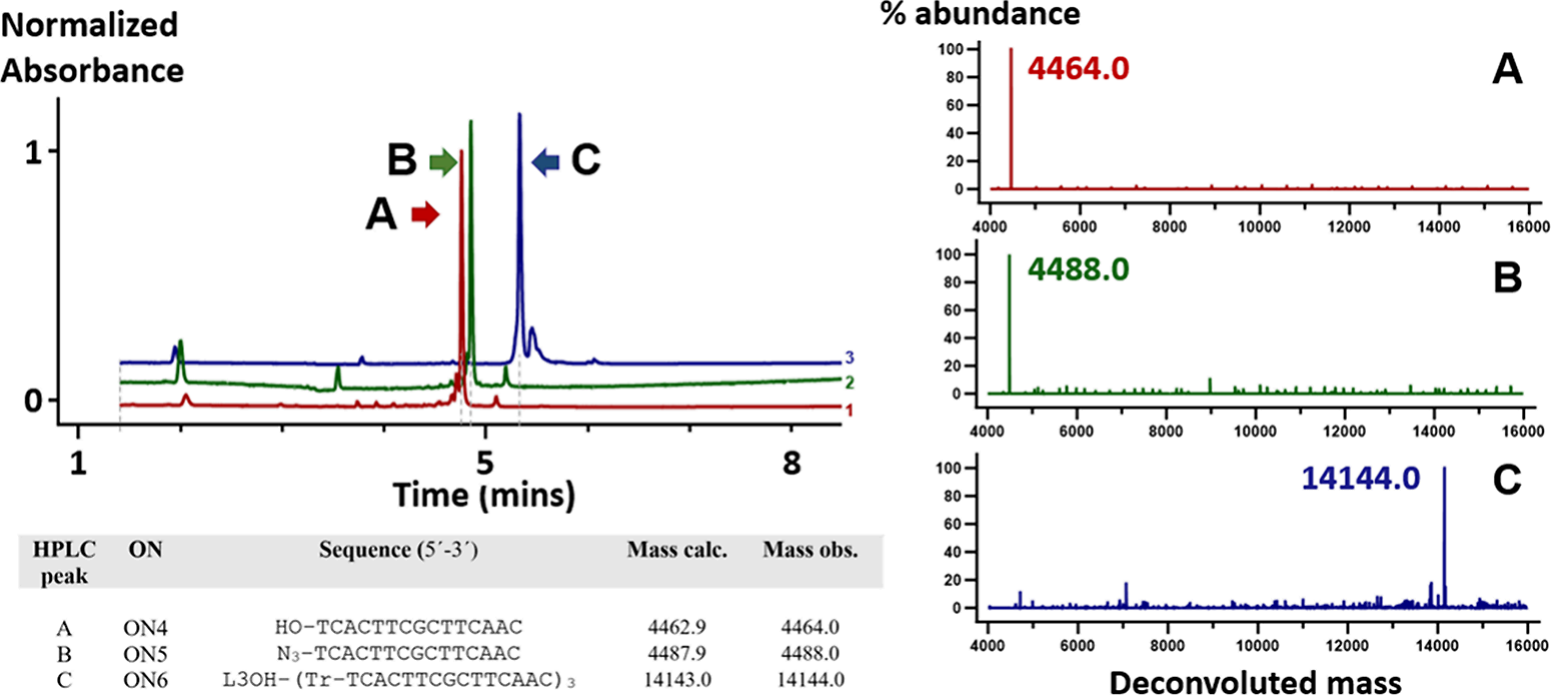
UPLC–MS analysis of mixed-sequence dendron synthesis of (A) **ON4**, starting ON; (B) **ON5**, 5′-azido intermediate; and (C) **ON6**, triconjugated product. The deconvoluted mass of individual peaks was shown on the right. CuAAC conditions: 1.0 mM CuSO_4_, 2.0 mM THPTA, 5.0 mM sodium ascorbate, and 500 μM L3OH in 100 mM TEAA buffer (50% DMSO, total volume = 1000 μL) at 45 °C for 4 h. L3OH represents the linker motif. Tr represents the triazole group.

The satisfactory conversion to trimeric dendrons on a solid support led us to explore the possibility to form higher order dendrons. Since L3OH contains a terminal hydroxyl group, which can be converted to azide, it is feasible to conjugate three dendrons via click chemistry to afford nonameric dendrons (Figure 4). To investigate this postulate, we applied the preceding reaction conditions to **ON1** on CPG to afford trimeric dendron **ON3**. Treatment with MTPPI and NaN_3_ led to 94% conversion to azide product **ON10** (linker moiety abbreviated as L3N_3_), which was subjected to a second click reaction with a reduced stoichiometry of L3OH. Post-cleavage and deprotection analysis revealed a broad peak on UPLC–MS that contained two major oligonucleotide products: hexamer (with one unreacted alkyne) and nonamer (Figure 5). Their co-eluting nature on HPLC demanded analysis by polyacrylamide gel electrophoresis (PAGE) (Figure S5A). For the first click reaction, an intense band corresponding to the trimeric dendron was separated from a faint band for the dimer, which has higher electrophoretic mobility. In comparison, the sample from the second click reaction contained multiple bands. We excised and isolated the most intense band and identified the nonameric dendron **ON11** by mass; the remaining bands were assigned as incomplete conjugates or unreacted species ranging from the dimer to octamer.

**Figure 4:**
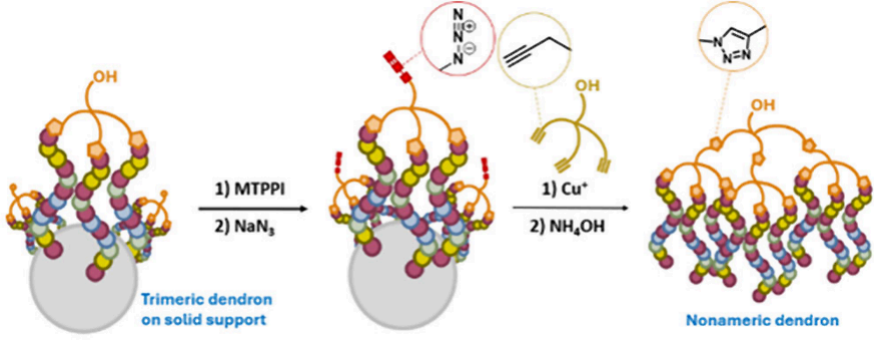
Synthesis of a nonameric dendron. The 5′-hydroxy groups of L3OH on trimeric dendrons are transformed into azido groups via treatment with MTPPI and sodium azide. This enables a second click reaction between three trimeric dendrons with L3OH to form a nonameric dendron.

**Figure 5:**
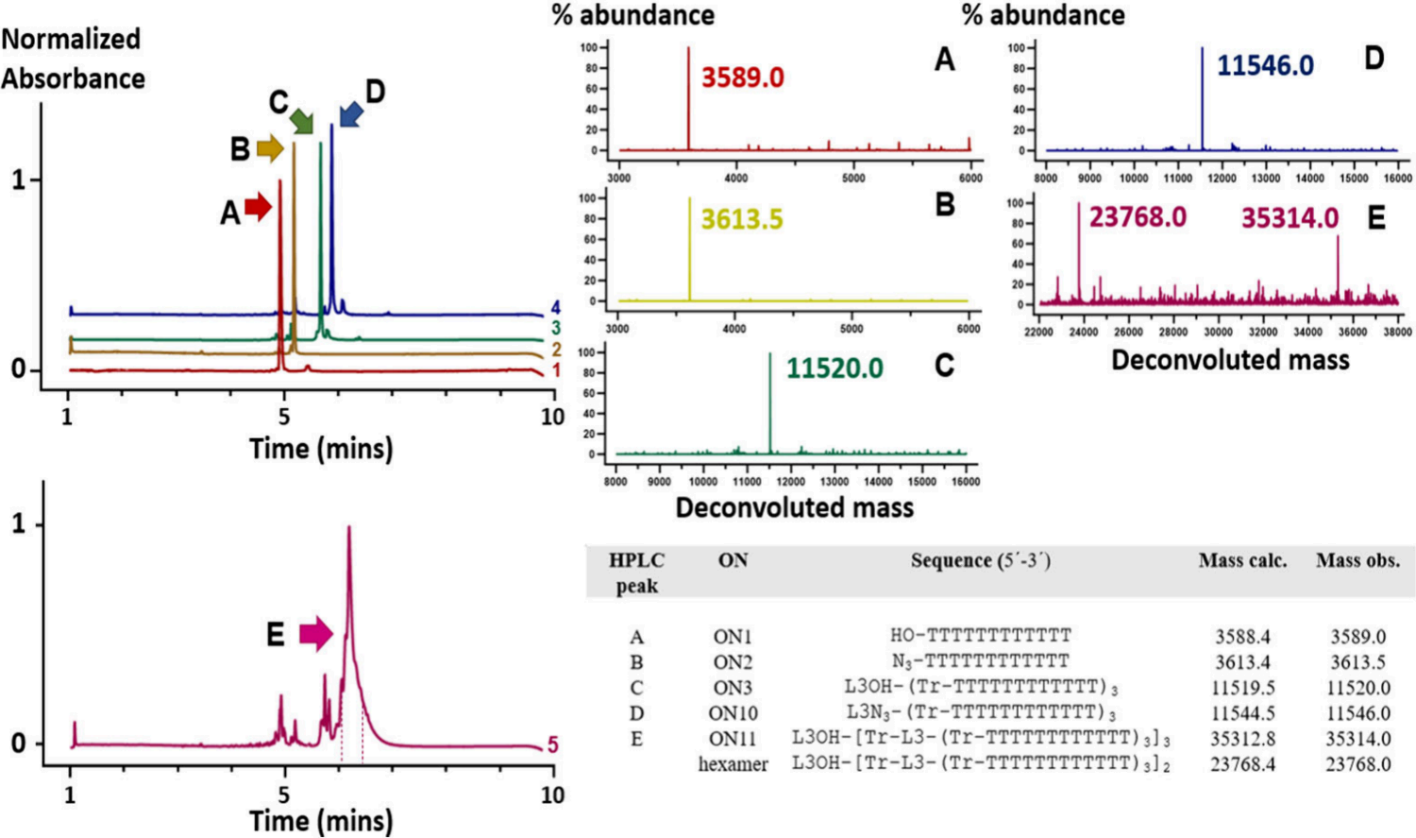
UPLC–MS data from nonameric NAD synthesis. Chemical transformations of **ON1** (HO-T_12_) to **ON10** (L3N_3_-(T_12_)_3_) in crude mixtures are indicated by HPLC peaks A–D. The retention profile of the crude reaction mixture indicated the presence of nonameric **ON11** and hexameric dendron side product in HPLC peak E. Deconvoluted masses of HPLC peaks A–E are visualized.

The presence of dendron conjugates of various valencies indicated that incomplete dendrons or unreacted oligonucleotide from the first click reaction could be converted to azide either at its 5′-hydroxy or on the hydroxy linker and participate in the second click reaction.

We then studied the biophysical and target-binding properties of dendrons synthesized from the mixed base sequence **ON4**. Trimeric (**ON6**) and nonameric (**ON13**) dendrons were prepared using the click conditions described above except that the loading of L3OH was lowered to 200 μM in the second click because only one-third as many hydroxyl groups are present. Purification of trimer **ON6** was done by reverse-phase HPLC, and that of nonamer **ON13** was done by PAGE (Figure S5B), resulting in a single peak identified on LC–MS with the correct mass (Figure 6). We examined their duplex stabilities against complementary targets by UV-thermal melting (Figure S6 and Table S2). With the incubation of an equimolar DNA target (3 μM), the *T*_m_ value of the conjugated trimeric duplexes was 57.5 °C (Δ*T*_m_ = −0.7 °C vs simple control DNA duplex). A similar slight decrease in duplex stability was observed with the complementary RNA target (*T*_m_ = 57.1 °C and Δ*T*_m_ = −0.7 °C). These findings agree with the literature that trimeric siRNA duplexes exhibit similar melting properties (Δ*T*_m_ = −0.1 to −0.6 °C) against their single-stranded counterparts.^8^ However, when the valency increased to nine in **ON13**, the duplex stability dropped against both DNA and RNA targets, with a decrease in the melting temperature of 4.5 and 3.4 °C compared to simple control duplexes, respectively. This is presumably due to increasing steric hindrance, which impacts the accessibility of the target strand as the cluster grows. Moreover, the high local concentrations of oligonucleotide strands with their propensity to form secondary structures can compete with binding to the target. We envisage that the low duplex stability of nonameric dendrons could be corrected using locked nucleic acid (LNA), which increases the binding affinity. Interestingly, the local maximum of d*A*/d*T* in the melting of **ON13** is almost 40% lower in magnitude than in **ON4** and **ON6**, suggesting that only 60% of the complementary strand is bound to nonameric dendron at a low temperature due to steric crowding. This hypothesis could be tested using a linker with longer arms to reduce the crowding effect.

**Figure 6:**
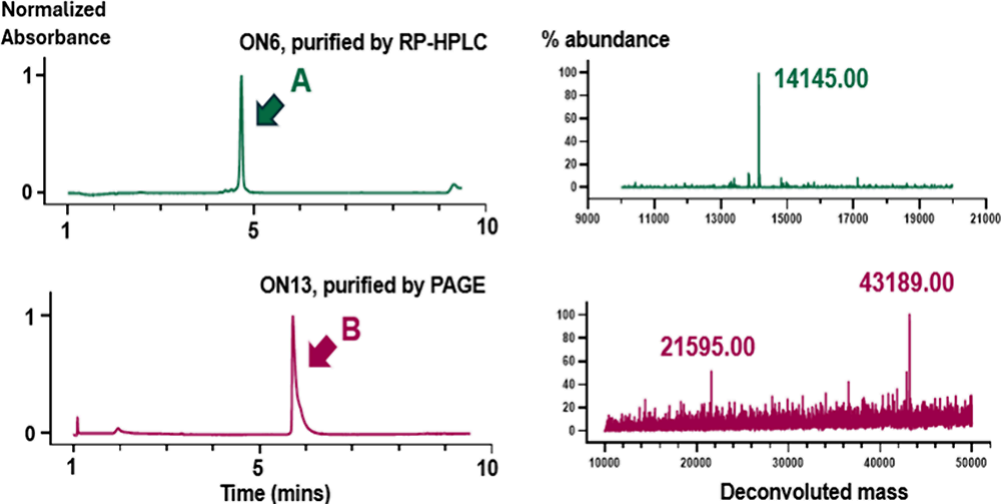
UPLC–MS analysis of dendrons **ON6** (trimer) and **ON13** (nonamer), purified by RP-HPLC and PAGE, respectively. **ON6** = L3OH-(Tr-TCACT­TCGCT­TCAAC)_3_, and **ON13** = L3OH-[Tr-L3-(Tr-TCACT­TCGCT­TCAAC)_3_]_3_. UPLC chromatograms and deconvoluted masses of the purified sample of **ON6** (calculated mass = 14 143.0) and **ON13** (calculated mass = 43 183.4) are shown.

We examined the ability of NADs to elicit RNase H cleavage, which is the principal mechanism to degrade target mRNA in antisense therapeutics. When complementary RNA substrate **ON14** with 5′-fluorescein (FAM) was incubated with **ON4** in the presence of RNase H, digestion was complete within 15 min. Similar digestion patterns were observed from the RNA co-incubated with **ON6** and **ON13** within 15 min (Figure S7). Hence, 5′-conjugation with multivalent linkers does not inhibit the RNase H activity. As expected, the dendrimers are digested by serum (Figure S8). For *in vivo* applications, this could be prevented by phosphorothioate and/or 2′-sugar modifications.

In summary, we report a facile method to synthesize NADs following standard SPOS and CuAAC click procedures, which eliminates the use of large excesses of reagents. Robust chemistry enables conversion rates up to 80% to the desired dendritic products and demonstrates versatility toward oligonucleotides with challenging modifications, such 2′-OTBDMS protecting groups. This method also enables the synthesis of higher order dendrons with up to nine oligonucleotide strands. Trimeric dendrons exhibit duplex stability with complementary RNA comparable to that of simple unmodified duplexes, whereas duplex stability drops with nonameric dendrons. Nevertheless, all constructs can elicit RNase H activity. This comparability in target-binding affinity, particularly with trimeric dendrons, raises the possibility of using NADs in a therapeutic context, such as RNase H-mediated mRNA degradation, steric blockers for exon skipping, or constructing siRNA and aptamer scaffolds.^8,30^ With their high molecular weights (up to 50 kDa), they are less prone to renal clearance^31,32^ and can potentially act as antisense therapeutic modalities with improved pharmacological properties. Prior to *in vivo* applications, it will be important to analyze the copper content of the constructs (e.g., by IPC–MS),^33,34^ as copper is toxic to cells.

## Supplementary Material



## Data Availability

The data underlying this study are available in the published article and its Supporting Information.

## Notes

Scheme S1, Tables S1 and S2, Figures S1–S8, experimental procedures, analytical data of synthesized compounds, ^1^H and ^13^C NMR spectra of novel compounds, and UPLC–HRMS of oligonucleotides (PDF)

## Notes

The authors declare that a patent application has been filed: Dendrimers, Patent Application 2509915.1, Oxford University Innovation, June 20, 2025.
