# Supplementary material for: New Approach to Assembling Nucleic Acid Dendrons on a Solid Phase
Source: Org Lett. 2025 Aug 20;27(34):9500–5. doi: 10.1021/acs.orglett.5c02877 (PMC12400419; doi:10.1021/acs.orglett.5c02877)
Supplement: Supplementary file 1 [file ol5c02877_si_001.pdf]

Supporting Information

A New Approach to Assembling Nucleic Acid Dendrons on Solid-Phase

Gerald J. Chik[a], Afaf H. El-Sagheer[a,b*], and Tom Brown[a*]

[a] Department of Chemistry, University of Oxford, Chemistry Research Laboratory, Oxford, OX1 3TA, UK.

[b] School of Chemistry, University of Southampton, Highfield, Southampton, SO17 1BJ, UK.

*Corresponding authors

*E-mail: : tom.brown@chem.ox.ac.uk and ahes@soton.ac.uk

Table of Contents

1. General information 2
2. Chemical Synthesis 3
3. Synthesis of oligonucleotides 5
4. List of oligonucleotides 8
5. Supplementary experimental data 9
6. UV melting experiments 14
7. RNase H activation assay 15
8. Foetal bovine serum stability assay 15
9. ^{1}H and ^{13}C NMR spectra for unreported compounds 17
10. References 20

1. General information

All reagents were purchased from Sigma-Aldrich, Acros Organics, Fisher Scientific, FluoroChem, Jena Bioscience or Alfa Aesar and used without further purification. Dry solvents (pyridine, Et_3N, CH_2Cl_2, THF, MeCN) were obtained using an MBraun SPS Bench Top solvent purification system (SPS). All air/moisture sensitive reactions were carried out under inert atmosphere (argon) in oven-dried glassware. Reactions were monitored by thin layer chromatography (TLC) using Merck Kieselgel 60 F24 silica gel plates (0.22 mm thickness, aluminium backed). The compounds were visualized by UV irradiation at 254/265 nm and by staining in p-anisaldehyde or ninhydrin solution followed by gentle heating on a hotplate. Flash column chromatography was performed using Merck Geduran® 60 Å (40–62 µm) or using a Biotage Sfär column. ^{1}H NMR (400 MHz, 500 MHz or 600 MHz), ^{13}C NMR (101 MHz, 126 MHz or 151 MHz) and ^{31}P NMR (162 MHz) spectra were measured on a Bruker AVIIIHD 400, a Bruker AVII 500 (with a ^{13}C cryoprobe) or a Bruker AVIII 600 (with a prodigy N2 broadband cryoprobe) spectrometer. ^{1}H and ^{13}C NMR spectra were referenced to the appropriate deuterated solvent signal, chemical shifts are given in ppm. Assignment of the NMR signals was aided by correlation spectroscopy (COSY; ^{1}H–^{1}H), heteronuclear single quantum correlation (HSQC; ^{1}H–^{13}C) and heteronuclear multiple bond correlation (HMBC; ^{1}H–^{13}C) experiments in specific experiments. Data was then processed using MestreNova® software. High resolution mass spectrometry (HRMS) using electrospray ionisation (ESI+) was recorded on a Thermo Exactive High-Resolution Orbitrap FTMS. Data was then deconvoluted using MassLynx v4.1.

2. Chemical Synthesis

Compound 4b[1]

To a flask charged with gallic acid, **4a** (6.00 g, 35.3 mmol) dissolved in methanol (125 mL) was added concentrated sulfuric acid (5 mL, 93.3 mmol). The solution was stirred at 80 °C under reflux for 18 hours. At this point, the solvent was removed under reduced pressure and the crude product was re-dissolved in ethyl acetate (150 mL) and washed with saturated sodium bicarbonate (150 mL). The product was further extracted from the aqueous phase with ethyl acetate (150 mL × 2). The combined organic phase was dried over $MgSO_4$ and in vacuo to afford compound **4b** (5.68 g, 87%) as an off-white solid. ^{1}H NMR (400 MHz, DMSO) δ 9.16 (s, 3H), 6.95 (s, 2H), 3.75 (s, 3H).

Compound 5a

To a flask charged with compound **4b** (460 mg, 2.5 mmol) and potassium carbonate (1.73 g, 12.5 mmol, 5 equiv.) was added N,N-dimethylformamide (12 mL). The suspension was stirred and heated up to 60 °C. To the mixture was added 3-(2-(2-Iodoethoxy)ethoxy)prop-1-yne (3.18 g, 12.5 mmol) dropwise over 45 minutes. The mixture was left stirring at 60°C for 18 hours and then concentrated under reduced pressure and resuspended in water/brine mixture (1:1 v/v) (120 mL). The crude product was extracted with dichloromethane (100 mL × 3), dried over $MgSO_4$, concentrated *in vacuo* and purified by column chromatography (SiO_2, EtOAc/Petroleum ether 50% to 70%) to afford compound **5a** (1.05 g, 75%) as an oil. Rf (EtOAc/Petroleum ether 70%) = 0.50. ^{1}H NMR (600 MHz, DMSO) δ 7.25 (s, 2H), 4.17 – 4.12 (m, 12H), 3.84 (s, 3H), 3.79 – 3.75 (m, 4H), 3.71 – 3.68 (m, 2H), 3.64 (dd, *J* = 5.9, 3.4 Hz, 4H), 3.61 – 3.57 (m, 6H), 3.55 (dd, *J* = 5.7, 3.3 Hz, 2H), 3.39 (q, *J* = 2.3 Hz, 3H). ^{13}C NMR (151 MHz, DMSO) δ 166.3, 152.5, 142.4, 124.9, 108.6, 80.8, 80.8, 77.5, 77.5, 72.4, 70.4, 70.1, 69.9, 69.4, 69.0, 69.0, 58.0, 58.0, 52.6. HRMS (ESI-TOF): calculated m/z: 563.2487 ([M+H]+, $C_{29}H_{39}O_{11}$+); found m/z: 563.2486.

Compound 5b

To a solution of compound **5a** (2.90 g, 5.15 mmol) in a mixture of dioxane / water (60 mL, 4:1 v/v) was added 4M aqueous sodium hydroxide (2.58 mL, 10.3 mmol, 2 equiv.). The mixture was stirred at 50 °C for 4 hours and then cooled down to room temperature. To the stirring mixture was added 1M hydrochloric acid (c.a. 11 mL) to acidify the mixture to pH 2.0. After the removal of dioxane under reduced pressure, the crude product was extracted from 1M hydrochloric acid (100 mL) with dichloromethane (100 mL × 3), dried over $MgSO_4$ and *in vacuo* to afford compound **5b** (2.85 g, quant.) as a pale yellow oil. ^{1}H NMR (600 MHz, DMSO) δ 12.86 (s, 1H), 7.23 (s, 2H), 4.17 – 4.10 (m, 12H), 3.78 – 3.75 (m, 4H), 3.71 – 3.68 (m, 2H), 3.64 (dd, *J* = 5.8, 3.4 Hz, 4H), 3.62 – 3.58 (m, 6H), 3.57 – 3.54 (m, 2H), 3.40 (t, *J* = 2.4 Hz, 3H). ^{13}C NMR (151 MHz, DMSO) δ 166.8, 151.8, 141.5, 125.6, 108.2, 80.3, 80.3, 77.0, 77.0, 71.9, 69.9, 69.6, 69.4, 68.9, 68.5, 68.4, 66.3, 57.5. HRMS (ESI-TOF): calculated m/z: 549.2330 ([M+H]+, $C_{28}H_{37}O_{11}$+); found m/z: 549.2324.

Compound 6 (L3OH)

To a solution of compound **5b** (291 mg, 0.53 mmol) in anhydrous *N,N*-dimethylformamide (10 mL) were added DIPEA (174 µL, 3 mmol) and HBTU (212 mg, 0.58 mmol, 1.1 equiv.). The mixture was stirred at room temperature for 30 minutes under argon atmosphere with gradual deepening of colour. Then, 2-(2-(2-aminoethoxy)ethoxy)ethanol (87 mg, 0.58 mmol, 1.1 equiv.) in anhydrous *N,N*-dimethylformamide (2 mL) was added to the mixture dropwise over 5 minutes. The mixture was stirred at room temperature for 24 hours, at which point the solvent was removed under reduced pressure. The crude product was re-suspended in water (30 mL), extracted with dichloromethane (30 mL × 3), washed with brine (50 mL × 1), dried over $MgSO_4$ and concentrated *in vacuo*. Column chromatography (SiO_2, MeOH/DCM 5% to 10%) afforded compound **6** (606 mg, 85%) as an oil. Rf (MeOH/DCM 10%) = 0.40. ^{1}H NMR (500 MHz, DMSO) δ 8.46 (t, *J* = 5.7 Hz, 1H), 7.18 (s, 2H), 4.57 (s, 1H), 4.14 (dd, *J* = 4.9, 2.4 Hz, 10H), 4.08 – 4.05 (m, 2H), 3.78 – 3.75 (m, 4H), 3.69 – 3.66 (m, 2H), 3.65 – 3.62 (m, 4H), 3.61 – 3.50 (m, 15H), 3.47 (d, *J* = 5.7 Hz, 2H), 3.42 – 3.38 (m, 6H). ^{13}C NMR (126 MHz, DMSO) δ 166.2, 152.4, 141.2, 130.1, 107.8, 80.9, 80.9, 77.0, 77.0, 72.9, 72.5, 70.4, 70.3, 70.3, 70.2, 70.0, 69.6, 69.3, 69.2, 60.9, 58.1, 31.8. HRMS (ESI-TOF): calculated m/z: 680.3277 ([M+H]+, $C_{34}H_{50}NO_{13}$+); found m/z: 680.3262.

3. Synthesis of oligonucleotides

I. Solid-phase synthesis of oligonucleotides

Standard DNA and 2′-OTBDMS protected RNA phosphoramidites, solid supports and reagents were purchased from Sigma Aldrich, Link Technologies and Applied Biosystems. Automated solid phase synthesis of oligonucleotides (trityl off) was performed on an Applied Biosystems 394 synthesiser using a standard phosphoramidite cycle of detritylation, coupling, oxidation and capping, at 0.2 or 1.0 µmole scale. Pre-packed nucleoside SynBase™ CPG 1000/110 synthesis resin (30 – 40 µmol/g, Link Technologies) was used, and β-cyanoethyl phosphoramidite monomers were dissolved in anhydrous MeCN to a concentration of 0.1 M immediately prior to use with a coupling time of 60 seconds (DNA) or 10 minutes (RNA). Stepwise coupling efficiencies and overall synthesis yields were determined by automated trityl cation conductivity monitoring facility and were >98% in all cases. The oligonucleotides were detritylated on solid support after the final coupling cycle.

II. Cleavage and deprotection from solid support

To cleave the oligonucleotides and nucleic acid dendrons from solid support with removal of nucleobase and backbone protecting groups, the resin was exposed to conc. NH_4OH for 60 minutes at room temperature followed by heating in a sealed glass vial in a heating block at 55 °C for 5 hours (DNA) or 2 hours (RNA). In the case of 2′-OTBDMS-protected RNA oligonucleotides, the solution sample was concentrated under reduced pressure until formation of a turbid solution and lyophilised overnight. The residue was then resuspended in a mixture of DMSO (300 µL) and $Et_3N{\cdot}3HF$ (300 µL) and incubated at 65 °C for 2.5 hours. Next, sodium acetate (3 M, 50 µL) and n-butanol (3 mL) were added to the sample and the solution was stored at –80 °C for a minimum of 1 hour. After thawing the suspension was pelleted under centrifuge (13,000 rpm, 4°C, 10 minutes) and the supernatant decanted. The precipitate was then washed with 70% v/v ethanol (2 × 750 µL), centrifuged at identical speed and then dried under vacuum. The crude oligonucleotides in solution were then concentrated *in vacuo*, re-dissolved in water and desalted using NAP™-10 column (G.E. Healthcare) prior to downstream lyophilisation or purification.

III. 5'-Azide conversion of oligonucleotides on solid support

After solid-phase synthesis, the resin-bound 5′-detritylated oligonucleotides contained in pre-packed columns were treated with methyltriphenoxyphosphonium iodide (0.5 M) in anhydrous DMF (1 mL) for 1 hour at room temperature. The solid support was then washed with dry DMF (3 × 1 mL) and dry MeCN (1 mL) and dried with argon. A saturated solution of sodium azide was then prepared by heating sodium azide (100 mg) in dry DMF (2 mL) for 5 minutes at 70 °C in a

sealed vial in a heating block. After cooling to room temperature, the resin was treated with the azide solution for 5 hours at 55 °C. The resin was then washed with DMF (3 × 1 mL) and MeCN (1 mL) and dried with argon. The 5′-azido oligonucleotides were cleaved from solid support and deprotected as described previously.

IV. Copper-catalysed click-crosslinking of 5′-azido oligonucleotides

To form trimeric dendrons, the resin containing the 5′-azido oligonucleotides (1 μmol) was washed with dry MeCN (1 mL) and dried thoroughly with argon. In the optimised click conditions, a click-reaction solution was prepared in a vial by mixing L3OH (5 mM, 100 μL), TEAA buffer (1.0 M, pH 7.0, 100 μL), Cu-THPTA solution (10 mM: 20 mM mixture, 100 μL), DMSO (500 μL) and water (150 μL). Immediately prior to the reaction, the catalyst was activated by the addition of sodium ascorbate (100 mM, 50 μL). The mixture was then degassed under a gentle stream of argon for 30 seconds and transferred to a 1-mL syringe. An empty syringe was connected to one end of the synthetic column containing the resin, and the reaction mixture was then passed through the column by plunging the syringes back and forth for 10 times or until the resin was completely submerged (i.e., when no air bubbles were observed from the column). The reaction set-up was incubated in the oven at 45 °C for 4 hours and the resin was agitated by gentle plunging every 60 minutes. After the reaction, the mixture was cooled down to room temperature, washed with DMF (3 × 1 mL) and MeCN (3 × 1 mL) and dried under argon. The crosslinked oligonucleotides were cleaved from solid support and deprotected as described previously.

To form nonameric dendrons from the 1-μmol synthesis, the stoichiometry of L3OH was reduced because theoretically only 33% of reactive azide groups remain after the first click reaction. The resin containing the trimeric dendrons connected by $L3N_3$ was subjected to the same treatment by a different reaction solution containing L3OH (5 mM, 50 μL), TEAA buffer (1.0 M, pH 7.0, 100 μL), Cu-THPTA solution (10 mM: 20 mM mixture, 100 μL), DMSO (500 μL), water (200 μL) and sodium ascorbate (100 mM, 50 μL).

V. HPLC purification of oligonucleotides

After cleavage and deprotection, oligonucleotides and nucleic acid dendrons were purified on Agilent HPLC system via RP-HPLC methods. Phenomenex Kinetex EVO C18 column (5.0 μm particle size, 10 mm × 250 mm) was used with a gradient of MeCN in aq. triethylammonium acetate (TEAA) buffers (Buffer A: 0.1 M TEAA, pH 7.0; buffer B: 0.1 M TEAA, 50% v/v MeCN, pH 7.0). Buffer B was increased from 20 - 40% (single stranded oligonucleotides), 20% - 60% (trimeric dendron – **ON6**) over 30 min at a flow rate of 5 $mL\cdot min^{-1}$ at 60 - 75°C, and elution was monitored by UV absorption at 260 nm with a 10.0 mm or 0.3 mm flow cell. After HPLC purification, oligonucleotides were lyophilised then dissolved in water without desalting.

VI. Analysis of oligonucleotides

All oligonucleotides were characterised by negative-mode ultra-performance liquid chromatography (UPLC) mass spectrometry using Waters Xevo G2-XS Q-Tof mass spectrometer with Acquity UPLC system. The system is equipped with Acquity UPLC oligonucleotide BEH C18 column (particle size: 1.7 μm; pore size: 130 Å; column dimensions: 2.1 mm x 50 mm). Data were analysed using Waters MassLynx software v 4.1 or Waters UNIFI Scientific Information System software.

4. List of oligonucleotides

	Sequence (5'-3')	Mass calc.	Mass obs.
**ON1**	HO-TTTTTTTTTTTT	3588.4	3588.0
**ON2**	N_3-TTTTTTTTTTTT	3613.4	3613.0
**ON3**	L3OH-(Tr-TTTTTTTTTTTT)$_3$	11519.5	11520.0
**ON4**	HO-TCACTTCGCTTCAAC	4462.9	4464.0
**ON5**	N_3-TCACTTCGCTTCAAC	4487.9	4489.0
**ON6**	L3OH-(Tr-TCACTTCGCTTCAAC)$_3$	14143.0	14145.5
**ON7**	HO-ucacuucgcuucaac	4632.8	4633.0
**ON8**	N_3-ucacuucgcuucaac	4657.8	4659.0
**ON9**	L3OH-(Tr-ucacuucgcuucaac)$_3$	14652.7	14655.0
**ON10**	L3N_3-(Tr-TTTTTTTTTTTT)$_3$	11544.5	11545.5
**ON11**	L3OH-[Tr-L3-(Tr-TTTTTTTTTTTT)$_3$]$_3$	35312.8	35314.0
**ON12**	L3N_3-(Tr-TCACTTCGCTTCAAC)$_3$	14168.0	14168.8
**ON13**	L3OH-[Tr-L3-(Tr-TCACTTCGCTTCAAC)$_3$]$_3$	43183.4	43189.0
**ON14**	FAM-guugaagcgaaguga	5415.0	5417.1

Table S1. Sequences of oligonucleotides used in this study. Upper-case letters represent DNA nucleosides and lower-case letters represent RNA nucleosides. The 5′-modifier is indicated as a functional group. L3OH and L3N_3 represent the linker motif with a hydroxy and azido end, respectively. Tr represents triazole group.

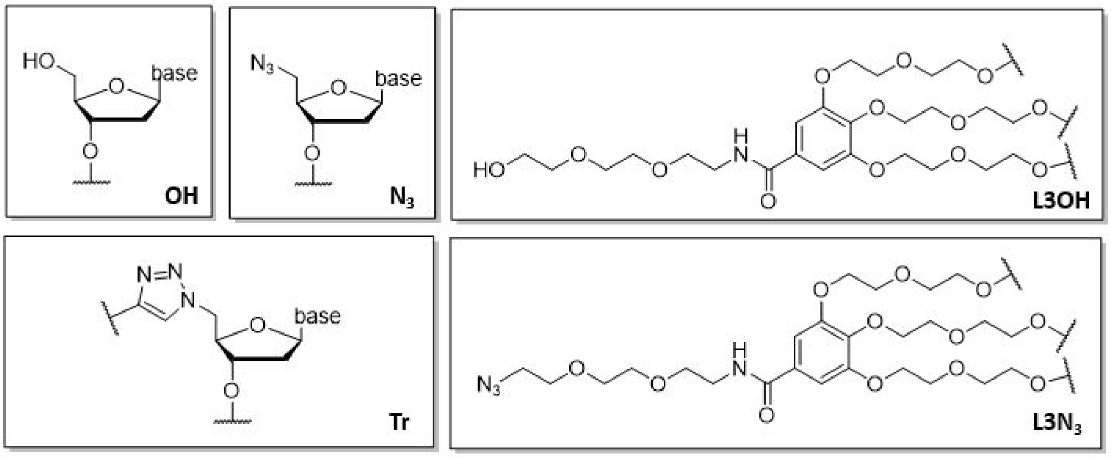

Figure S1. Chemical structures of 5'-Modifiers of oligonucleotides described in Table S1.

5. Supplementary experimental data

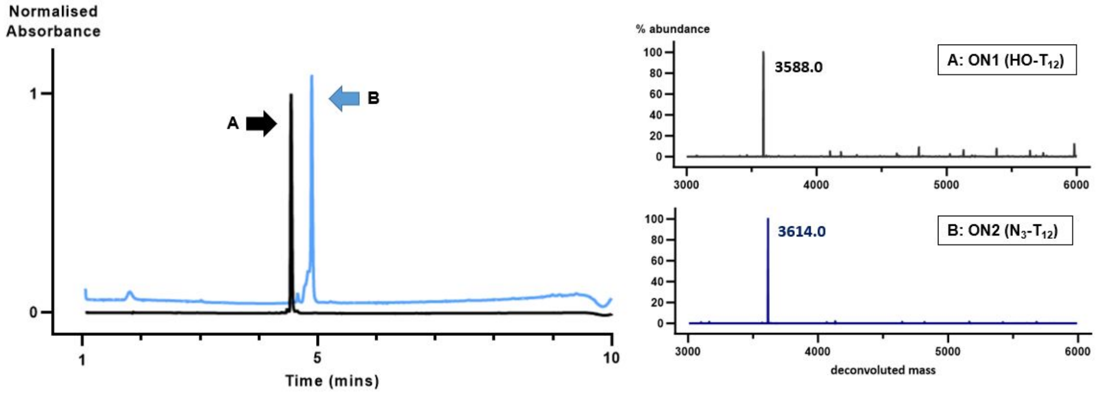

Figure S2. UPLC-MS results of 5’-hydroxy-to-azide conversion on ON1
Overlaid UPL chromatograms are shown on the left. Black line indicates the retention profile of ON1 (starting material HO-T_{12}, calc. mass = 3588.4). Blue line indicates that of ON2 (conversion product N_3-T_{12}, calc. mass = 3613.4) under standard azide conversion conditions: (i) 0.5 M MTPPI/DMF, r.t., 1 h; (ii) wash with dry DMF and MeCN then dry under Ar; (iii) sat. NaN_3/DMF, 55°C, 5 hours on UPLC-MS. Deconvoluted masses of individual peaks are shown on the right.

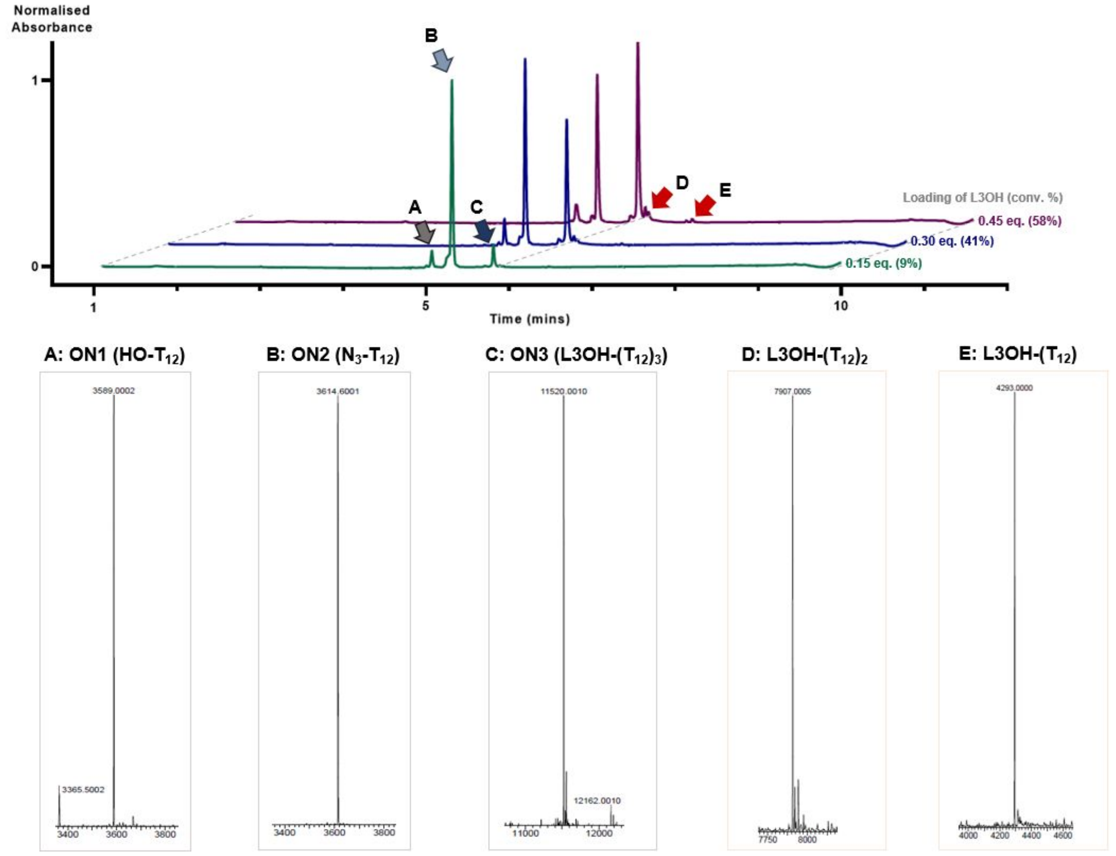

Figure S3. UPLC-MS results from CuAAC crosslinking with various eq. of L3OH

Stacked UPL chromatograms are shown on the top. General CuAAC condition: 0.5 mM $CuSO_4$, 1.0 mM THPTA, 1.0 mM sodium ascorbate in 100 mM TEAA buffer (20% DMSO, total volume = 200 µL), incubated at r.t. in an Eppendorf Thermomixer®, 24 hours. The three sets of experiment are loaded with various equivalents of L3OH (to a final conc. of 50, 100 and 150 µM). The retention profiles are indicated as green, blue and violet lines, respectively. Deconvoluted masses of the major peaks are shown at the bottom, assigned to a corresponding species: (A) **ON1** (HO-T_{12}), calc. mass = 3588.4; (B) **ON2** (N_3-T_{12}), calc. mass = 3613.4; (C) **ON3** (L3OH-$(T_{12})_3$), calc. mass = 11519.5; (D) L3OH-$(T_{12})_2$, calc. mass = 7906.1; and (E) L3OH-(T_{12}), calc. mass = 4292.7. Conversion % was calculated as the ratio between area under the product peak and that of starting material, product and by-product.

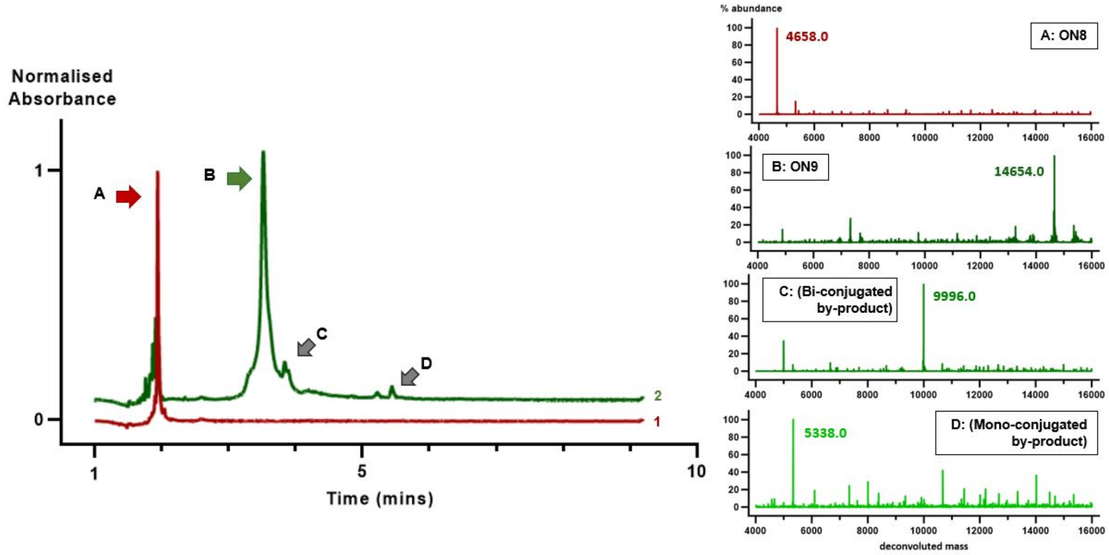

Figure S4. Crude UPLC-MS results from RNA dendron synthesis

Overlaid UPL chromatograms show the retention profiles of the crude sample of (A) **ON8**, 5'-azido intermediate (calc. mass = 4657.8); (B) **ON9**, tri-conjugated product (calc. mass = 14652.7). Deconvoluted mass of individual peaks was shown on the right. CuAAC crosslinking condition: 1.0 mM CuSO4, 2.0 mM THPTA, 5.0 mM sodium ascorbate, 500 μM L3OH in 100 mM TEAA buffer (50% DMSO, total volume = 1000 μL), 45°C, 4 hours.

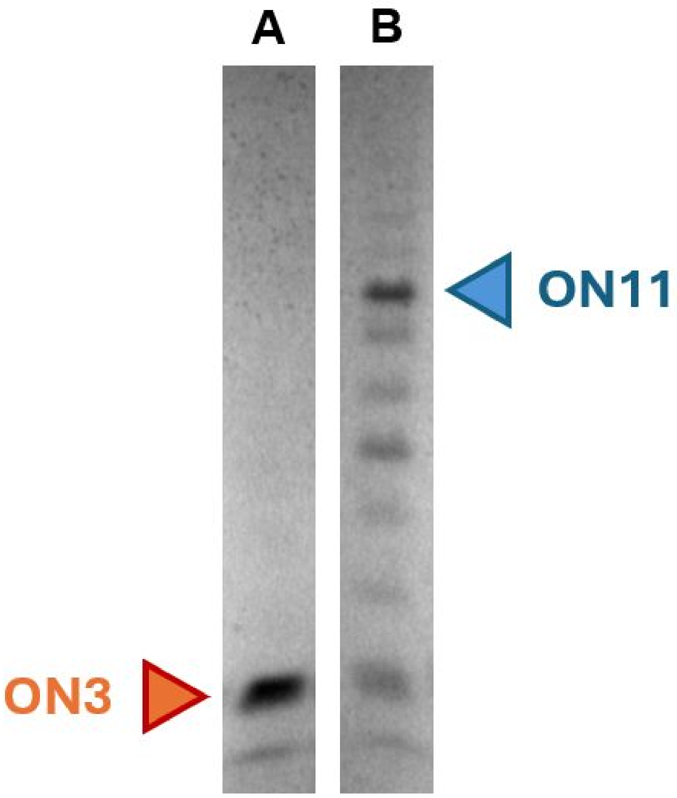

Figure S5A. PAGE-gel analysis of click reactions on T_{12} oligonucleotide
The results of 8% PAGE analysis of the mixtures from the first and second click reaction are excerpted from the same gel at identical height, as shown as **A** and **B**, respectively. Orange triangle indicates the major band as **ON3** and blue indicates that as **ON11**. Both bands were excised and extracted into water and analysed by UPLC-MS for mass confirmation.

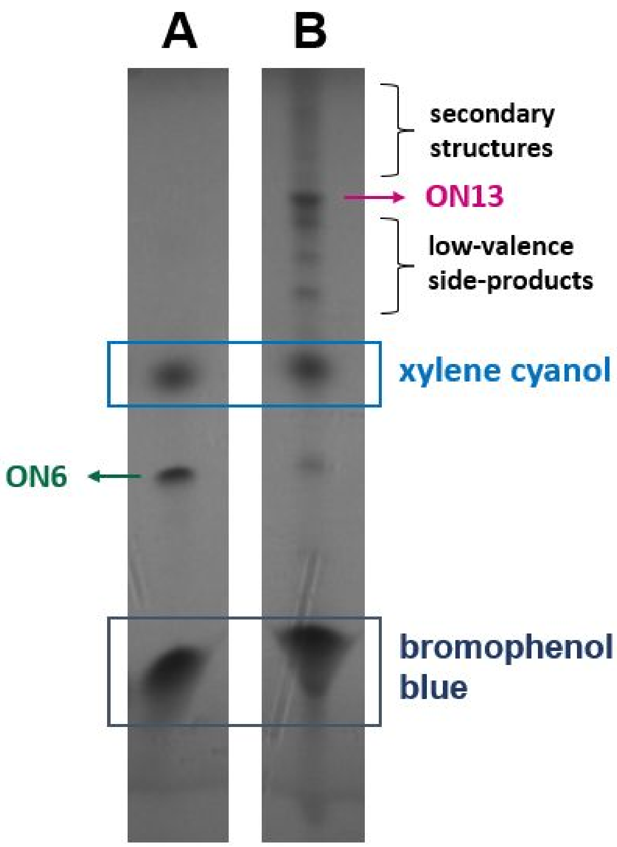

Figure S5B. PAGE-gel analysis of click reactions on oligonucleotide ON4
The results of 8% PAGE analysis of the mixtures from the first and second click reaction are excerpted from the same gel at identical height, as shown as **A** and **B**, respectively. Trimeric **ON6** and nonameric **ON13** were indicated by an arrow. Bands with lower electrophoretic mobility were postulated to be secondary structures and bands with higher electrophoretic mobility were postulated to be low-valence side products. Both product bands were excised and extracted into water and analysed by UPLC-MS for mass confirmation.

6. UV melting experiments

Experimental procedures

UV DNA melting curves were recorded on a Cary 4000 Scan UV-Visible Spectrophotometer using complementary oligonucleotides (3 µM each strand) in a 10 mM phosphate buffer containing 200 mM NaCl at pH 7.0. Samples were melted by heating in the instrument's Peltier block to 85°C (10 °C/min) and then annealed by slowly cooling to 20 °C (1 °C/min). Six successive cycles (including heating and cooling) were then performed at a gradient of 0.5 °C/min and the change in UV absorbance at 260 nm was recorded. The melting temperature of each experiment was calculated from a smoothed plot of dA/dT (first derivative of the melting curve) from six ramps using in built software.

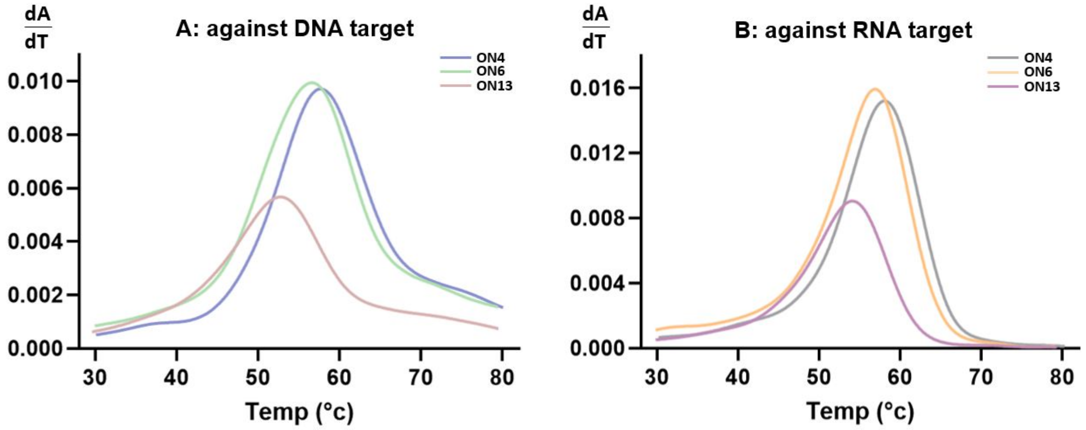

Figure S6. First derivative of UV-thermal melting curves of oligonucleotide dendrons
The two plots describe the first derivative *Tm* curves of oligonucleotide strand and dendrons (**ON4**, **ON6** and **ON13**) against DNA (A) and RNA (B) targets. Measurement was taken with 3 µM of oligonucleotides with equimolar DNA or RNA target in 10 mM NaH_2PO_4, 200 mM NaCl buffer (pH 7.0) and from 30–80°C. *Tm* values were determined from an average of six ramps from a smoothed plot of dA/dT.

Table S2

Oligonucleotide	Tm vs DNA deg C	Tm vs RNA deg C
ON4 (monomer)	58.2	57.8
ON6 (trimer)	57.5	57.1
ON13 (nonamer)	53.7	54.4

7. RNase H activation assay

Experimental procedures

Escherichia coli RNase H (NEB) was used for all RNase H studies. Oligonucleotides (0.4 µM for ON4, ON6 and ON13) and corresponding FAM-labelled RNA target ON14 (2.0 µM) and *E. coli* RNase H (20 U/mL) were incubated at 37 °C or 20 °C in 1 × RNase H reaction buffer (50 mM Tris-HCl, 75 mM KCl, 3 mM MgCl2, 10 mM dithiothreitol, pH 8.3). At each time point, 13 µL aliquots were taken and quenched with aq EDTA-Na_2 (2 µL, 500 mM) at a concentration of 66.7 mM, denatured with formamide 1/1 (v/v), snap-frozen and kept at –20 °C in the dark. The aliquots were submitted to a 20% PAGE (400 V) and analysed using a G:Box (*Syngene*) with an excitation wavelength of λ = 460 nm and an emission filter of λ = 516–600 nm for detection of the FAM label.

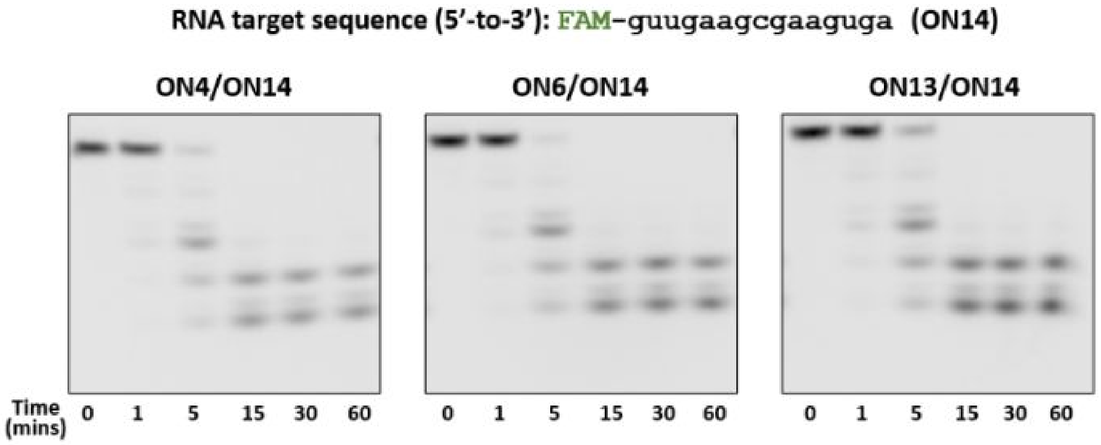

Figure S7. PAGE analysis of RNase H activation assay. RNA target sequence (**ON14**) is shown on the top. The three images show the electrophoretic migration of aliquots of RNA sample **ON14** (2.0 µM) when incubated with **ON4**, **ON6** and **ON13** (0.4 µM) together with *E. Coli* RNase H (20 U/mL) in RNase H reaction buffer® (50 mM Tris-HCl, 75 mM KCl, 3 mM $MgCl_2$, 10 mM DTT) sampled at different time interval over 60 mins at 37°C. The digestion pattern of **ON14** is visualised as fluorescent band (inverted) on the gel.

8. Foetal bovine serum stability assay

Nuclease resistance of trimeric dendron **ON6** and nonameric dendron **ON13** did not show significant difference when compared to single strand **ON4** when incubated in 50% FBS (Figure S7). In principle, nuclease resistance can be improved by incorporation of phosphorothioate linkages in the constituent oligonucleotides.

Experimental procedures

Oligonucleotides (ON4, ON6 and ON13; 5 nmole of single-stranded oligo) were dissolved in 50 µL of Dulbecco's PBS and 50 µL of FBS (Gibco). This was then vortexed and 20 µL were removed (t = 0 min; control), mixed with formamide 1/1 (v/v), snap-frozen and kept at −20 °C. The remaining solution was incubated at 37 °C and 20 µL aliquots were removed at specified time points, mixed with formamide 1/1 (v/v), snap-frozen and kept at −20 °C. Samples were analysed by PAGE (300 V) and viewed under short wavelength UV light using a G:Box (*Syngene*).

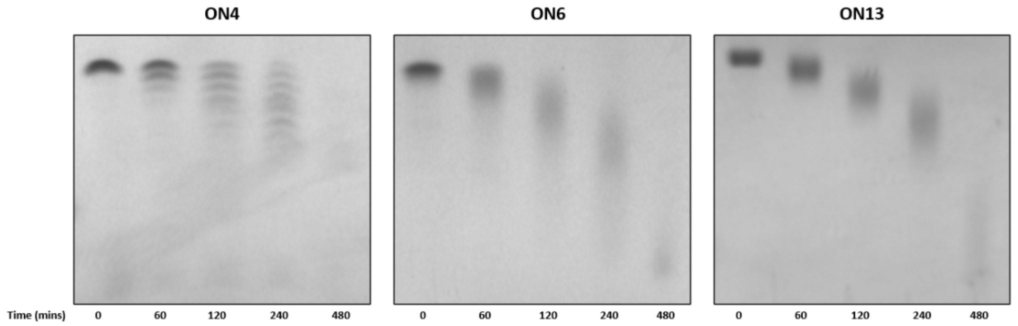

Figure S8. Results of PAGE analysis from FBS stability assay

The three images show the electrophoretic migration of the aliquots of samples **ON4**, **ON6** and **ON13** incubated in 50% v/v FBS/PBS solution (pH 7.4) over 0, 60, 120, 240 and 480 minutes at 37°C. The degradation pattern of each oligonucleotide species is shown. The % of polyacrylamide in the PAGE analysis of **ON4** = 20%, **ON6** =12% and **ON13** = 8% (left to right).

9. ^{1}H and ^{13}C NMR spectra for unreported compounds

Compound 5a

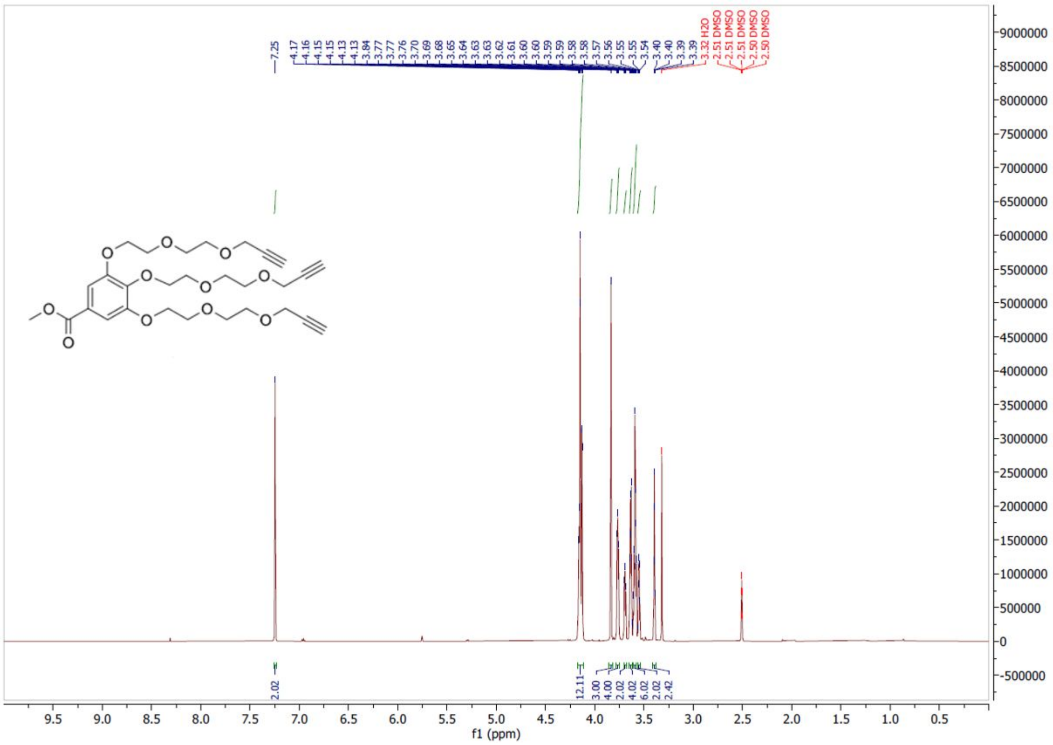

^{1}H NMR (400 MHz, *d*-DMSO) spectrum of **5a**

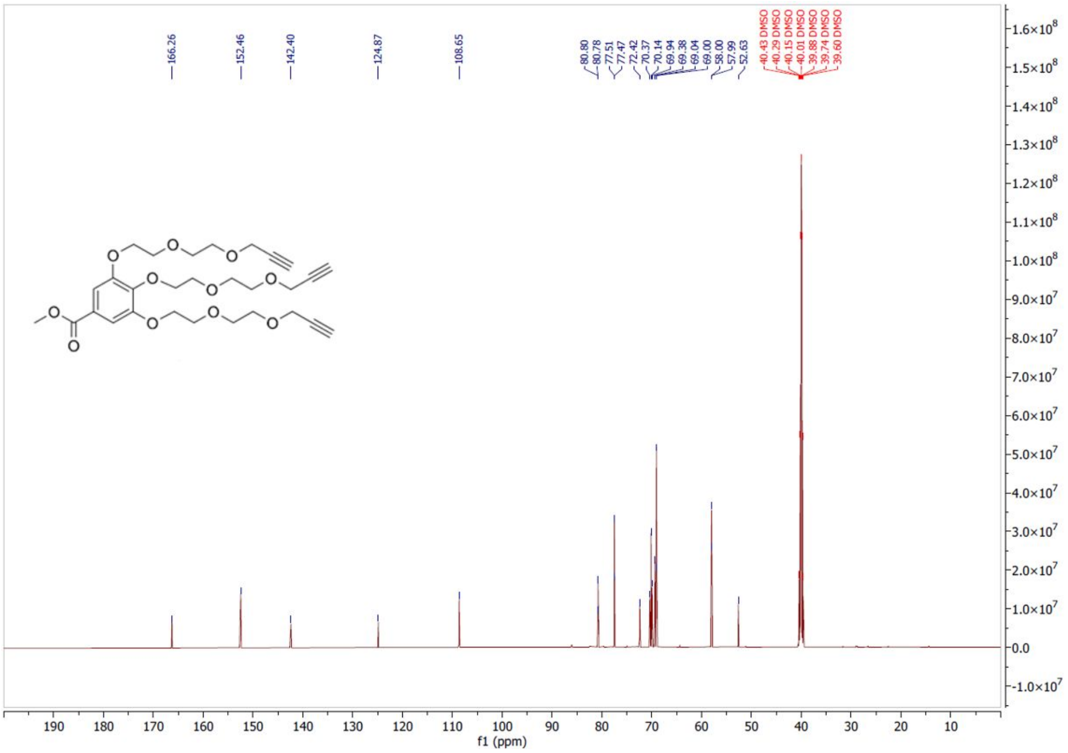

^{13}C NMR (101 MHz, *d*-DMSO) spectrum of **5a**

Compound 5b

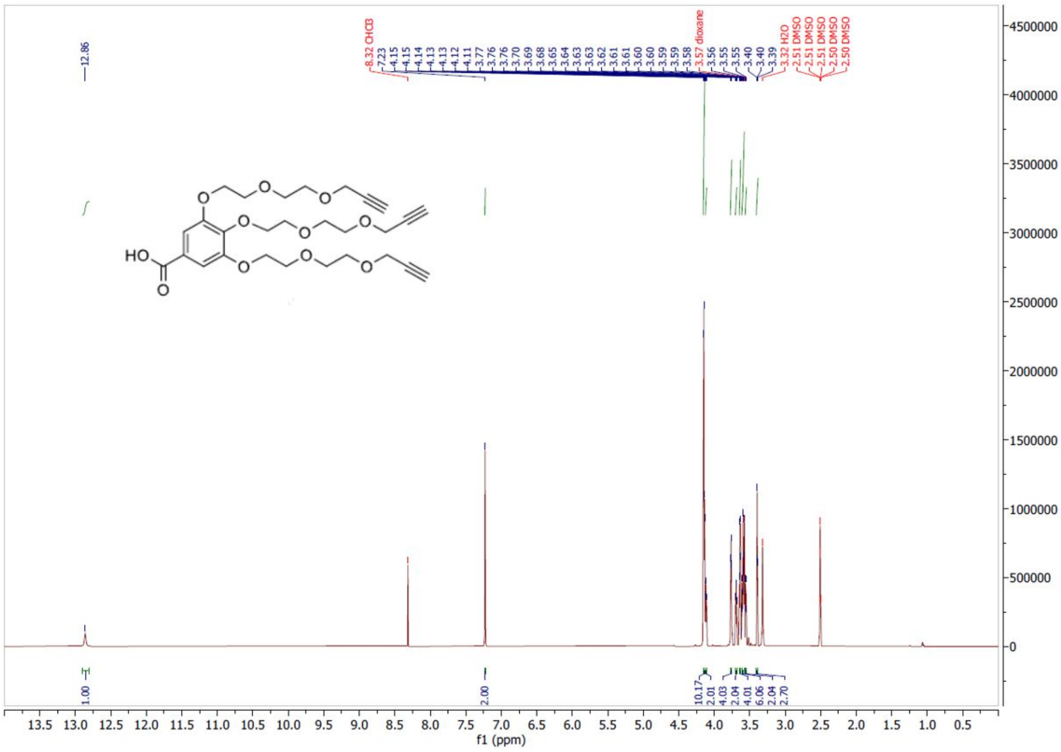

^{1}H NMR (400 MHz, *d*-DMSO) spectrum of **5b**

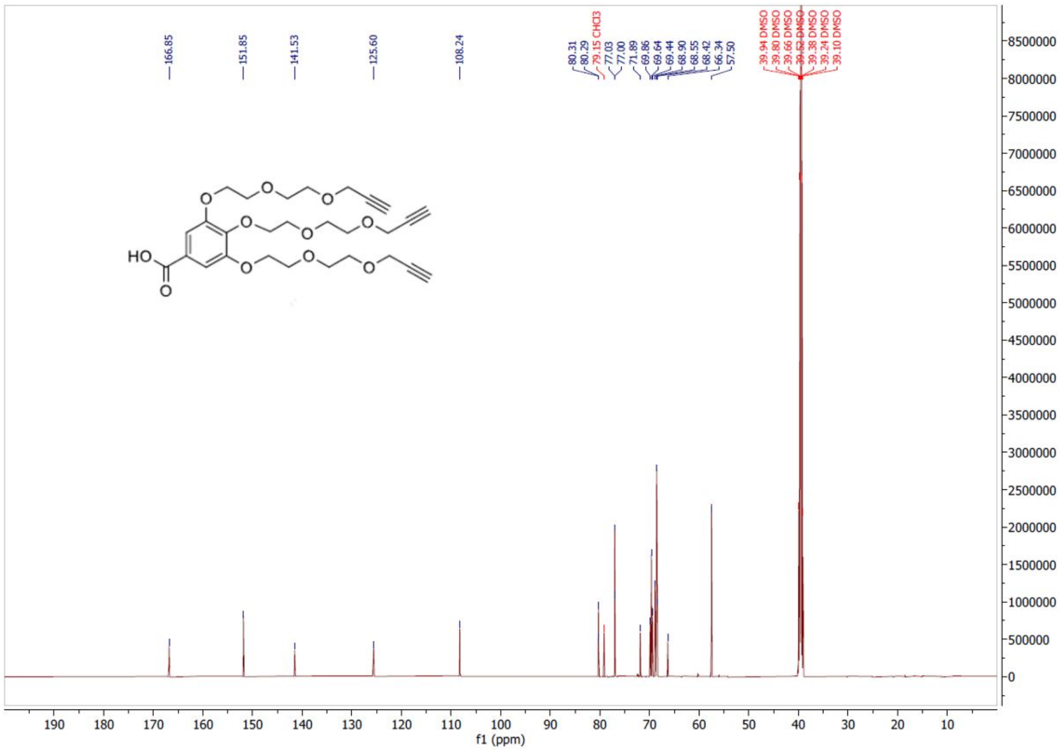

^{13}C NMR (101 MHz, *d*-DMSO) spectrum of **5b**

Compound 6

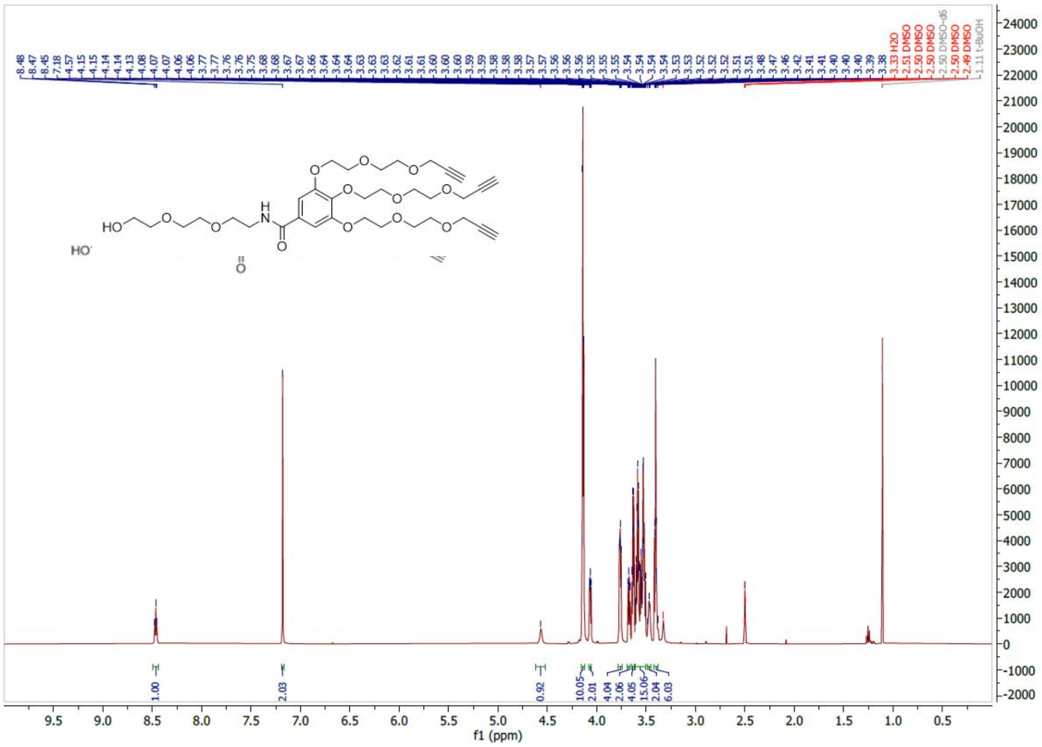

^{1}H NMR (400 MHz, *d*-DMSO) spectrum of **6**

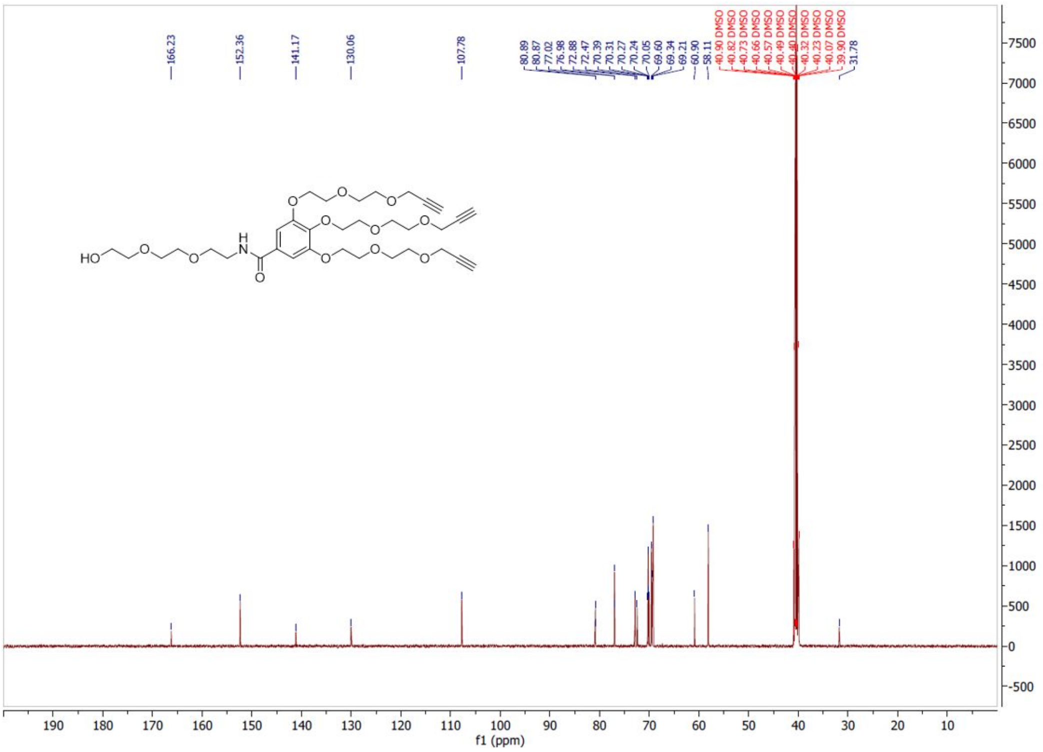

^{13}C NMR (101 MHz, *d*-DMSO) spectrum of **6**

10. References

(1) Fukuda, T.; Matsumoto, E.; Onogi, S.; Miura, Y. Aggregation of Alzheimer Amyloid β Peptide (1-42) on the Multivalent Sulfonated Sugar Interface. *Bioconjug Chem* 2010, *21* (6), 1079–1086.
